# Identification of Diagnostic and Prognostic Subnetwork Biomarkers for Women with Breast Cancer Using Integrative Genomic and Network-Based Analysis

**DOI:** 10.3390/ijms252312779

**Published:** 2024-11-28

**Authors:** Olfat Al-Harazi, Achraf El Allali, Namik Kaya, Dilek Colak

**Affiliations:** 1 Molecular Oncology Department, King Faisal Specialist Hospital and Research Centre, Riyadh 11211, Saudi Arabia; 2 Bioinformatics Laboratory, College of Computing, Mohammed VI Polytechnic University, Benguerir 43150, Morocco; 3 Translational Genomics Department, Center for Genomic Medicine, King Faisal Specialist Hospital and Research Centre, Riyadh 11211, Saudi Arabia

**Keywords:** breast cancer, network, biomarker, omics, prediction, subnetwork

## Abstract

Breast cancer remains a major global health concern and a leading cause of cancer-related deaths among women. Early detection and effective treatment are essential in improving patient survival. Advances in omics technologies have provided deeper insights into the molecular mechanisms underlying breast cancer. This study aimed to identify subnetwork markers with diagnostic and prognostic potential by integrating genome-wide gene expression data with protein–protein interaction networks. We identified four significant subnetworks revealing potentially important hub genes, including *VEGFA*, *KIF4A*, *ZWINT*, *PTPRU*, *IKBKE*, *STYK1*, *CENPO*, and *UBE2C*. The diagnostic and prognostic potentials of these subnetworks were validated using independent datasets. Unsupervised principal component analysis demonstrated a clear separation of breast cancer patients from healthy controls across multiple datasets. A KNN classification model, based on these subnetworks, achieved an accuracy of 97%, sensitivity of 98%, specificity of 94%, and area under the curve (AUC) of 96%. Moreover, the prognostic significance of these subnetwork markers was validated using independent transcriptomic datasets comprising over 4000 patients. These findings suggest that subnetwork markers derived from integrated genomic network analyses can enhance our understanding of the molecular landscape of breast cancer, potentially leading to improved diagnostic, prognostic, and therapeutic strategies.

## 1. Introduction

Breast cancer is the most frequently diagnosed cancer and remains the leading cause of cancer-related mortality among women worldwide [1]. There is a growing need for precise diagnostic and prognostic tools that can capture the molecular complexity of breast cancer and guide personalized treatment strategies [2,3]. High-throughput genomic technologies have revolutionized breast cancer research by enabling the identification of gene signatures associated with distinct subtypes and clinical outcomes, thus providing valuable insights into underlying molecular mechanisms [4,5,6]. Recent studies suggest that integrating biological context and network interactions between genes can lead to more robust biomarkers and improve our understanding of complex cancer biology [7,8,9,10].

Network-based approaches provide a systems biology perspective by integrating gene expression data with protein–protein interaction (PPI) networks to identify subnetworks functionally linked to specific disease states [7,11]. These subnetworks, also known as disease modules, represent groups of interconnected genes or proteins involved in shared biological processes and pathways associated with a disease state [7,8,12]. Subnetwork markers derived from integrative analyses have been shown to be more robust and reliable for disease diagnosis and prognosis than individual gene markers, as they capture the complex gene interactions and regulatory networks driving tumorigenesis [8,9,10,13,14,15,16].

This study aimed to identify subnetwork biomarkers for breast cancer through the integration of genome-wide gene expression data with PPI networks. Clustering algorithms were applied to partition the gene network into distinct subnetworks and activity scores for each subnetwork were computed. These scores were then used to develop a KNN-based classification model. The performance of this model was validated using an independent dataset from The Cancer Genome Atlas (TCGA). Moreover, we assessed the prognostic significance of the identified subnetworks through survival analyses using independent transcriptomic datasets from diverse ethnic and racial populations. The results suggest that subnetwork markers derived from integrated network analyses may provide a comprehensive understanding of breast cancer’s molecular architecture, potentially leading to improved diagnostics and prognostics across populations.

## 2. Results

### 2.1. Identification of Subnetwork Biomarkers

**Figure 1:**
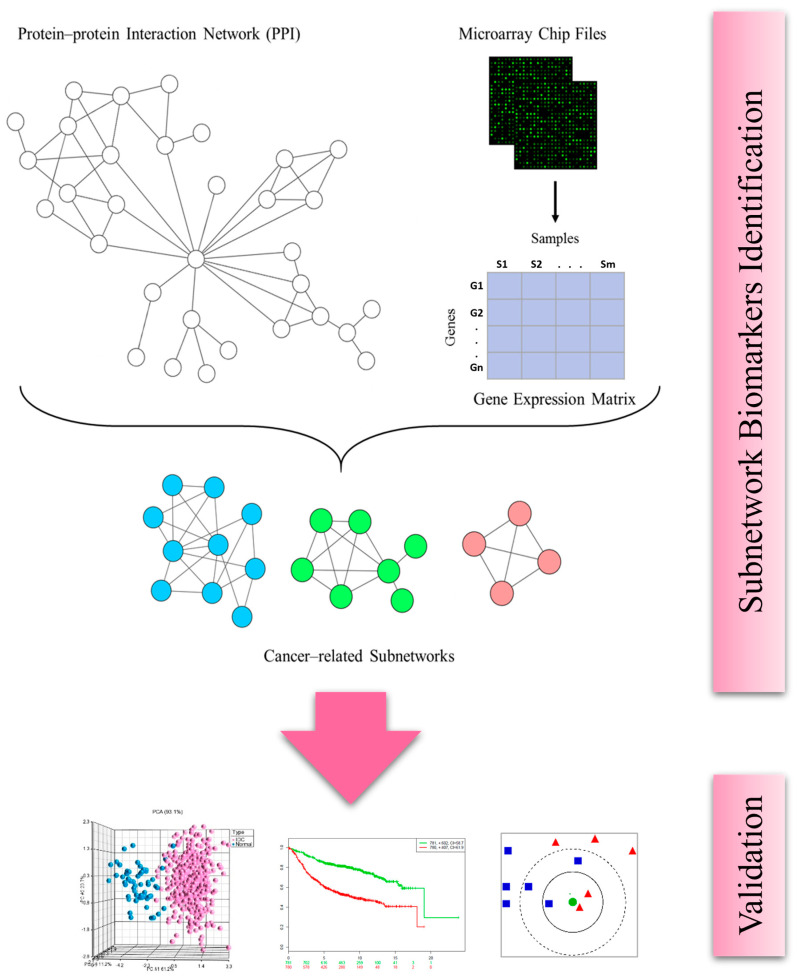
Schematic flowchart illustrating the methodology.

**Figure 2:**
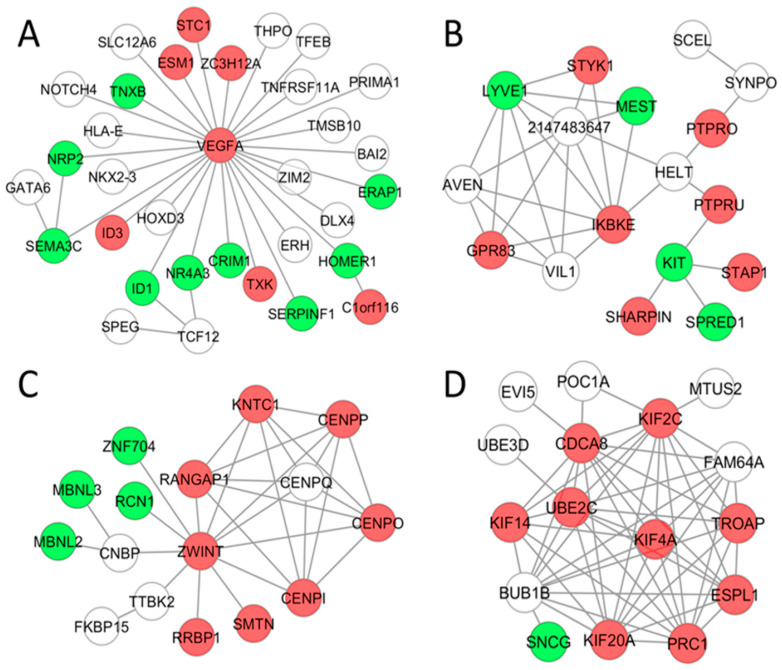
Four significant subnetworks associated with breast cancer: (**A**) Subnetwork 1, (**B**) Subnetwork 2, (**C**) Subnetwork 3, and (**D**) Subnetwork 4. Red nodes indicate over-expressed genes in breast cancer and green nodes indicate under-expressed ones.

We integrated gene expression data with protein–protein interaction (PPI) networks to identify disease-specific molecular modules or subnetworks. The PPIs were extracted from the STRING database [17]. The Context-Specific Subnetwork Discovery (COSSY) algorithm was employed to cluster the gene network into subnetworks, as detailed in the methods section [18,19] (Figure 1). To assess the significance of these subnetworks, Welch’s *t*-tests were performed to compare subnetwork activity scores between invasive ductal carcinoma (IDC) samples and normal controls. The analysis identified four subnetworks exhibiting statistically significant differences in activity between IDC and control samples (adjusted *p*-value < 0.00001, corrected using the Benjamini–Hochberg method), suggesting distinct patterns of dysregulation in cancerous tissues. The identified subnetwork biomarkers revealed potentially important hub genes, including VEGFA, KIF4A, ZWINT, PTPRU, IKBKE, STYK1, CENPO, and UBE2C (Figure 2).

Functional enrichment analyses of genes within each subnetwork using the Database for Annotation, Visualization, and Integrated Discovery (DAVID) bioinformatics tool (DAVID version 6.8) [20] revealed significant enrichment of biological processes related to angiogenesis, cell migration, the regulation of cell proliferation, chromatin organization, cell division, and cell cycles (Table 1).

### 2.2. Diagnostic Significance of the Identified Subnetwork Biomarkers

**Figure 3:**
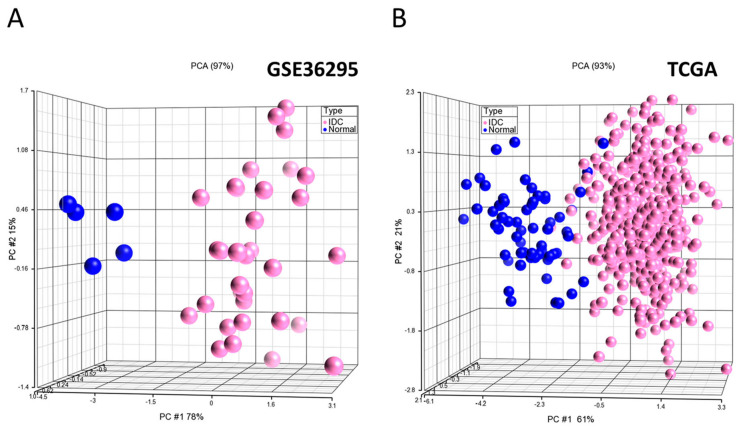
Unsupervised principal component analysis (PCA) of samples from GSE36295 (*n* = 39 samples) (**A**) and TCGA (*n* = 522 samples) (**B**) using the identified subnetwork biomarkers. Pink spheres indicate tumor; blue spheres indicate normal. The PCA analysis clearly distinguished breast cancer patients from normal controls.

To evaluate the diagnostic value of the identified subnetwork biomarkers, we performed unsupervised principle component analysis (PCA) on two datasets: GSE36295 and TCGA (Figure 3A and Figure 3B, respectively). In the PCA scatter plots, each sphere denotes a sample in the datasets. The PCA analysis clearly separated samples as BC patients (pink spheres) and normal controls (blue spheres) in both datasets (Figure 3).

Subsequently, a KNN classifier was constructed using the four subnetwork markers, with the GSE36295 dataset used for training. Subnetwork activity scores were computed for each sample and the classifier was trained using the city block distance measure with k = five neighbors. A two-level nested cross-validation approach was utilized to ensure robust model validation, as detailed in the methods section. The classification performance was then evaluated, which showed perfect discrimination between tumor samples and normal controls on the training dataset and an accuracy of 97%, sensitivity of 98%, specificity of 94%, and AUC of 96% on the TCGA dataset.

### 2.3. Prognostic Significance of the Identified Subnetwork Biomarkers

**Figure 4:**
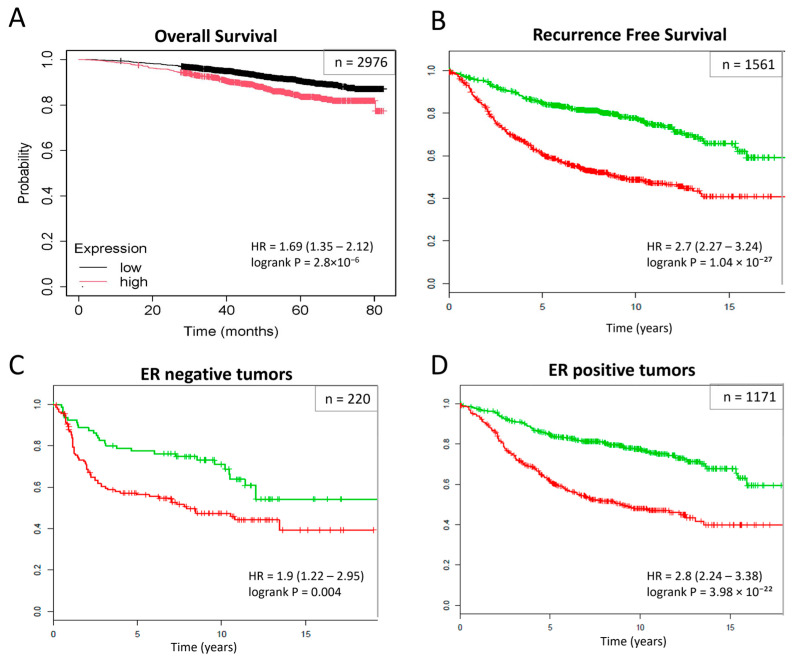
Prognostic significance of subnetwork markers. Overall survival (OS) analysis of breast tumor samples in Dataset 1 (*n* = 2976 samples in GSE96058) (**A**), recurrence-free survival (RFS) analysis of breast tumor samples in Dataset 2 (*n* = 1561) (**B**), and patients stratified into ER (−) and ER (+), respectively (**C**,**D**). Kaplan–Meier curves for risk groups; patients with high scores (“high-risk group”) had significantly lower recurrence-free survival compared to the low-risk group.

The prognostic significance of the subnetwork biomarkers was assessed for overall survival (OS) and recurrence-free survival (RFS) using two large transcriptomic datasets. High subnetwork activity scores were found to be significantly associated with poor clinical outcomes (*p*-value < 0.0001) (Figure 4A and Figure 4B, respectively). Further stratification of patients based on estrogen receptor (ER) status revealed that these subnetwork markers could effectively distinguish between high-risk and low-risk groups within both ER-negative and ER-positive cohorts (*p*-value < 0.01) (Figure 4C and Figure 4D, respectively).

## 3. Discussion

In this study, we identified four significant subnetwork biomarkers with diagnostic and prognostic potential in breast cancer through the integration of gene expression data with PPI networks. These subnetworks demonstrated robust diagnostic and prognostic capabilities, consistent with previous findings that emphasize the reliability of network-based methodologies in identifying functionally relevant biomarkers and potential therapeutic targets across various cancers [8,9,10,16].

The integration of gene expression data with PPI networks enabled a more comprehensive understanding of breast cancer’s molecular landscape. The identified subnetworks revealed key significantly dysregulated hub genes, including *VEGFA*, *KIF4A*, *ZWINT*, *PTPRU*, *IKBKE*, *STYK1*, *CENPO*, and *UBE2C*, which play crucial roles in various biological processes associated with cancer progression, such as angiogenesis, cell cycle regulation, and mitotic nuclear division. For example, *VEGFA* (Vascular Endothelial Growth Factor A) is a well-known mediator of angiogenesis, critical for tumor growth and metastasis. The overexpression of *VEGFA* has been associated with poor prognosis in multiple cancers, including breast cancer, due to its role in promoting angiogenesis and enhancing the metastatic potential of tumor cells [21]. Similarly, *KIF4A*, *ZWINT*, and *UBE2C* are involved in cell cycle regulation, cell proliferation, and mitosis, with frequent dysregulation observed in cancers and a clear association with tumor progression and poor clinical outcomes [6,20,22,23,24,25]. *PTPRU*, *IKBKE*, *STYK1*, and *CENPO* are implicated in key biological pathways relevant to cancer biology, including cell signaling, cell migration, and inflammation, further supporting their relevance in tumorigenesis [26,27]. These findings suggest that the identification of markers through a network-based approach may lead to key driver genes that are involved in tumorigenesis and highlight associated pathways that could represent a promising therapeutic strategy.

The high accuracy, sensitivity, specificity, and AUC of the KNN classification model based on these subnetworks confirm their potential as reliable diagnostic markers. Moreover, the significant prognostic utility of these subnetworks for overall survival (OS) and recurrence-free survival (RFS) in large patient cohorts emphasizes their clinical relevance. Furthermore, the ability to distinguish between high-risk and low-risk groups within both ER-negative and ER-positive cohorts further supports the potential of these subnetwork markers in guiding personalized treatment strategies.

Despite the promising results, several limitations of this study must be acknowledged. First, the analysis predominantly relied on data from Saudi patients (GSE36295), and the sample size was relatively small, which may have potentially introduced bias and limited the generalizability of the findings across different ethnic and socioeconomic groups. Nevertheless, validation results on larger cohorts, including TCGA and Swedish datasets, illustrated the diagnostic and prognostic potential of the identified subnetwork markers, suggesting their robustness across populations. These findings support the hypothesis that subnetwork markers derived from network-based approaches are more stable across diverse populations than traditional single-gene markers due to their reliance on systems biology approaches. This stability can be particularly advantageous in global health contexts, where biomarkers need to perform consistently across various genetic and environmental backgrounds.

Future research should focus on validating subnetwork biomarkers in diverse populations and exploring their potential in predicting responses to various treatments, including targeted therapies and immunotherapies. Moreover, integrating additional omics data, such as proteomics, metabolomics, and single-cell sequencing data, could further enhance our understanding of the molecular landscape of breast cancer and better capture tumor heterogeneity. Such comprehensive approaches may lead to the discovery of additional biomarkers and therapeutic targets, ultimately advancing precision medicine in breast cancer diagnosis, prognosis, and treatment.

## 4. Materials and Methods

### 4.1. Gene Expression Datasets

Whole-genome gene expression data for Saudi patients with breast cancer (Invasive Ductal Carcinoma, IDC) were obtained from the NCBI Gene Expression Omnibus (GEO) www.ncbi.nlm.nih.gov/geo (accessed on 23 July 2018) under accession number GSE36295, which includes 39 samples (34 tumors and 5 normal controls), processed on the Affymetrix Human Gene 1.0 ST Array platform. Additionally, the gene expression dataset for IDC patients from The Cancer Genome Atlas (TCGA) was downloaded, consisting of 460 tumor samples and 62 normal controls.

### 4.2. Subnetwork Identification

Gene expression data were integrated with protein–protein interaction (PPI) networks to identify significant subnetwork biomarkers using the Context-Specific Subnetwork Discovery (COSSY) algorithm [18,19]. Molecular interaction networks were retrieved from the Search Tool for the Retrieval of Interacting Genes/Proteins (STRING) database (Version 10.5) [17] and partitioned into subnetworks using the iCOSSY online platform http://icossy.korea.ac.kr/ (accessed on 26 July 2018) [18]. Each subnetwork represented a cluster of closely interacting molecular nodes. Subnetworks were visualized using Cytoscape version 3.4.0 [28].

The COSSY algorithm partitions an interaction network into smaller, closely connected subnetworks using a non-greedy approach. It ranks these subnetworks based on the expression patterns of their respective genes, assigning an entropy score to indicate their importance. Lower scores correspond to higher significance. The algorithm selects the five most differentially expressed genes for each subnetwork using a modified Welch’s *t*-statistic based on the interquartile range (IQR). If multiple subnetworks significantly overlap, they are merged to create a new subnetwork. The COSSY algorithm then clusters samples using the expression values of the representative genes and calculates an entropy score based on the distribution of different sample classes in each cluster [18,19].

### 4.3. Classification

Activity scores for each subnetwork were calculated by subtracting the average expression of down-regulated genes from the average expression of up-regulated genes for each sample. These scores were then used as feature values to construct classification models, using the GSE36295 dataset as the training set. A K-nearest neighbor (KNN) algorithm was employed for classifier construction, with a 2-level nested cross-validation approach (10 outer and 10 inner partitions) ensuring robust model validation. The KNN classifier was trained using the city block distance measure with k = 5 neighbors. Classification performance was evaluated on the TCGA dataset (460 tumors and 62 normal controls) by assessing accuracy, sensitivity, specificity, and AUC [9].

### 4.4. Survival Analysis

Kaplan–Meier survival analyses were performed using the identified subnetwork biomarkers on two transcriptomic datasets: GSE96058 (Dataset 1; *n* = 2976 breast cancer samples) [29,30] and an additional dataset (Dataset 2; *n* = 1561) [29,30,31]. The significance between survival curves was calculated using the log-rank test, with the statistical significance threshold set at *p*-value < 0.05.

## 5. Conclusions

In conclusion, this study identified novel subnetwork biomarkers for breast cancer with diagnostic and prognostic potential. The integration of gene expression data with PPI networks offers a systems biology approach that captures the complexity of molecular interactions underlying breast cancer. These findings lay the foundation for developing more precise diagnostic, prognostic, and therapeutic strategies for various cancers, thereby contributing to improved patient outcomes. Future research should focus on validating these subnetwork biomarkers across diverse populations, integrating additional omics data and exploring their utility in predicting responses to various treatments, including targeted therapies and immunotherapies.

## Figures and Tables

**Table 1:** Gene Ontology (GO) enrichment analysis of four subnetworks.

GO Biological Process Term	*p*-Value	Genes
**Subnetwork 1**		
blood vessel development	1.8 × 10^−7^	*NRP2*, *SLC12A6*, *SERPINF1*, *ID1*, *GATA6*, *NOTCH4*, *VEGFA*, *ZC3H12A*, *SEMA3C*, *ESM1*
angiogenesis	2.0 × 10^−7^	*NRP2*, *SLC12A6*, *SERPINF1*, *ID1*, *GATA6*, *NOTCH4*, *VEGFA*, *ZC3H12A*, *ESM1*
gland development	3.6 × 10^−6^	*TNFRSF11A*, *SERPINF1*, *GATA6*, *HOXD3*, *NOTCH4*, *VEGFA*, *SEMA3C*, *NKX2-3*
regulation of cell proliferation	1.4 × 10^−5^	*NRP2*, *TNFRSF11A*, *SERPINF1*, *ID1*, *SPEG*, *GATA6*, *VEGFA*, *TXK*, *ESM1*, *NR4A3*, *NKX2-3*, *THPO*
cell surface receptor signaling pathway	1.8 × 10^−5^	*NRP2*, *ERH*, *NR4A3*, *ESM1*, *HOMER1*, *TNFRSF11A*, *ID1*, *GATA6*, *HOXD3*, *NOTCH4*, *VEGFA*, *SEMA3C*, *TXK*, *CRIM1*, *THPO*
**Subnetwork 2**		
cell surface receptor signaling pathway	5.2 × 10^−4^	*GPR83*, *STYK1*, *STAP1*, *SHARPIN*, *VIL1*, *SPRED1*, *PTPRU*, *KIT*, *PTPRO*
enzyme-linked receptor protein signaling pathway	7.9 × 10^−4^	*STYK1*, *STAP1*, *VIL1*, *SPRED1*, *PTPRU*, *KIT*
cell migration	2.1 × 10^−3^	*STYK1*, *STAP1*, *VIL1*, *PTPRU*, *KIT*, *PTPRO*
movement of cell or subcellular component	2.2 × 10^−3^	*STYK1*, *LYVE1*, *STAP1*, *VIL1*, *PTPRU*, *KIT*, *PTPRO*
cell motility	3.5 × 10^−3^	*STYK1*, *STAP1*, *VIL1*, *PTPRU*, *KIT*, *PTPRO*
**Subnetwork 3**		
sister chromatid cohesion	8.7 × 10^−10^	*CENPO*, *CENPQ*, *ZWINT*, *CENPP*, *KNTC1*, *RANGAP1*, *CENPI*
CENP-A-containing chromatin organization	7.1 × 10^−6^	*CENPO*, *CENPQ*, *CENPP*, *CENPI*
DNA replication-independent nucleosome assembly	1.4 × 10^−5^	*CENPO*, *CENPQ*, *CENPP*, *CENPI*
histone exchange	1.9 × 10^−5^	*CENPO*, *CENPQ*, *CENPP*, *CENPI*
single-organism organelle organization	2.5 × 10^−4^	*CENPO*, *TTBK2*, *CENPQ*, *ZWINT*, *CENPP*, *KNTC1*, *RANGAP1*, *CENPI*
**Subnetwork 4**		
cell division	3.4 × 10^−12^	*KIF14*, *KIF2C*, *FAM64A*, *KIF4A*, *CDCA8*, *PRC1*, *EVI5*, *ESPL1*, *UBE2C*, *KIF20A*
mitotic nuclear division	2.2 × 10^−9^	*KIF14*, *KIF2C*, *FAM64A*, *KIF4A*, *CDCA8*, *PRC1*, *ESPL1*, *UBE2C*
chromosome segregation	2.6 × 10^−8^	*KIF14*, *KIF2C*, *KIF4A*, *CDCA8*, *PRC1*, *ESPL1*, *UBE2C*
organelle fission	2.8 × 10^−8^	*KIF14*, *KIF2C*, *FAM64A*, *KIF4A*, *CDCA8*, *PRC1*, *ESPL1*, *UBE2C*
cell cycle	4.6 × 10^−8^	*KIF14*, *KIF2C*, *FAM64A*, *KIF4A*, *CDCA8*, *PRC1*, *EVI5*, *ESPL1*, *UBE2C*, *KIF20A*

## Data Availability

We used publicly available datasets in this study from The Cancer Genome Atlas (TCGA) and Gene Expression Omnibus (GEO).
